# Rectal bacteriome and virome signatures and clinical outcomes in community-acquired pneumonia: An exploratory study

**DOI:** 10.1016/j.eclinm.2021.101074

**Published:** 2021-08-12

**Authors:** Robert F.J. Kullberg, Floor Hugenholtz, Xanthe Brands, Cormac M. Kinsella, Hessel Peters-Sengers, Joe M. Butler, Martin Deijs, Michelle Klein, Daniël R. Faber, Brendon P. Scicluna, Tom Van der Poll, Lia Van der Hoek, W. Joost Wiersinga, Bastiaan W. Haak

**Affiliations:** a Center for Experimental and Molecular Medicine (CEMM), Amsterdam University Medical Centers - Location AMC, University of Amsterdam, Meibergdreef 9, Room G2-130, Amsterdam 1105 AZ, the Netherlands; b Laboratory of Experimental Virology, Department of Medical Microbiology and Infection Prevention, Amsterdam UMC, Location AMC, Amsterdam, the Netherlands; c Department of Internal Medicine, BovenIJ hospital, Amsterdam, the Netherlands; d Department of Clinical Epidemiology, Biostatistics and Bioinformatics, Amsterdam University Medical Centers - Location AMC, University of Amsterdam, Amsterdam, the Netherlands; e Division of Infectious Diseases, Amsterdam University Medical Centers - Location AMC, University of Amsterdam, Amsterdam, the Netherlands

**Keywords:** Community-acquired pneumonia, Intestinal microbiota, Virome, Clinical outcomes

## Abstract

Background Bacterial intestinal communities interact with the immune system and may contribute to protection against community-acquired pneumonia (CAP). Intestinal viruses are closely integrated with these bacterial communities, yet the composition and clinical significance of these communities in CAP patients are unknown. The aims of this exploratory study were to characterise the composition of the rectal bacteriome and virome at hospital admission for CAP, and to determine if microbiota signatures correlate with clinical outcomes.

Methods We performed a prospective observational cohort study in CAP patients, admitted to a university or community hospital in the Netherlands between October 2016 and July 2018, and controls. Rectal bacteriome and virome composition were characterised using 16S ribosomal RNA gene sequencing and virus discovery next-generation sequencing, respectively. Unsupervised multi-omics factor analysis was used to assess the co-variation of bacterial and viral communities, which served as primary predictor. The clinical outcomes of interest were the time to clinical stability and the length of hospital stay.

Findings 64 patients and 38 controls were analysed. Rectal bacterial alpha (*p* = 0•0015) and beta diversity (*r^2^*=0•023, *p* = 0•004) of CAP patients differed from controls. Bacterial and viral microbiota signatures correlated with the time to clinical stability (hazard ratio 0•43, 95% confidence interval 0•20–0•93, *p* = 0•032) and the length of hospital stay (hazard ratio 0•37, 95% confidence interval 0•17–0•81, *p* = 0•012), although only the latter remained significant following *p*-value adjustment for examining multiple candidate cut-points (*p* = 0•12 and *p* = 0•046, respectively).

Interpretation This exploratory study provides preliminary evidence that intestinal bacteriome and virome signatures could be linked with clinical outcomes in CAP. Such exploratory data, when validated in independent cohorts, could inform the development of a microbiota-based diagnostic panel used to predict clinical outcomes in CAP.

Funding Netherlands Organization for Scientific Research and Netherlands Organization for Health Research and Development.

**Research in context**

**Evidence before this study**

Preclinical studies have shown that respiratory tract infections are associated with changes in the composition of gut microbiota, which in turn influence the function of the immune system against respiratory pathogens. These findings have yet to be confirmed in humans and their clinical significance in patients with community-acquired pneumonia (CAP) is unknown. We searched PubMed with the terms “(respiratory tract infections[mh] OR pneumonia[tiab]) AND (microbiota[mh] OR microbiome[tiab]) AND (case-control studies[mh] OR prospective[tiab])” for articles published in any language up to March 20, 2021. The search identified 150 results. There were no prospective studies assessing the effects of CAP on the intestinal bacteriome and virome in humans.

**Added value of this study**

This exploratory study is the first to characterise the rectal bacteriome and virome during CAP in humans. We show that alterations of the intestinal microbiota during CAP not only involve bacteria, but extend to the viral microbiota. Moreover, by using a validated unsupervised matrix factorisation analysis tool, we identified a gut microbiota signature - characterized by a loss of obligately anaerobic bacteria and Lactococcus phages combined with an enrichment of the bacterial genera *Staphylococcus* and *Enterococcus*, and their corresponding bacteriophages - which was independently associated with worse clinical outcomes in CAP patients.

**Implications of all the available evidence**

The intestinal microbiota has an important role in influencing the function of the immune system against respiratory infections. This proof-of principle study provides preliminary evidence that alterations of these communities are independently associated with clinical outcomes in CAP patients. These exploratory data, when validated in independent cohorts, could inform the development of a microbiota-based diagnostic panel used to predict disease severity and clinical outcomes in hospitalised patients.

Alt-text: Unlabelled box

## Introduction

Bacterial and viral pneumonia accounts for more deaths than any other infectious disease worldwide and survivors of community-acquired pneumonia (CAP) are at increased risk of long-term mortality and re-hospitalisation over subsequent months to years [1,2]. There is an urgent need for insight into the complex pathophysiological processes underlying pneumonia and its ensuing recovery.

The human intestinal microbiota is among the most complex microbial environments on earth and comprises bacteria, eukaryotic viruses, bacteriophages, fungi and archaea [3,4]. We and others have not only shown that the intestinal bacteriome is extensively altered during sepsis and intensive care unit (ICU) stay - characterised by a decrease in microbial diversity and an expansion of potentially pathogenic bacteria - but also that gut bacteria can influence the function of the immune system in distant organs [5], [6], [7]. For example, it has been shown in preclinical models that metabolites produced by obligate anaerobic gut bacteria, such as short-chain fatty acids, are capable of priming alveolar macrophages in the systemic circulation. This process enhances the activity of these macrophages against respiratory pathogens (e.g. *Streptococcus pneumoniae* and influenza virus) [8], [9], [10]. Furthermore, gut derived short-chain fatty acids (such as acetate and butyrate) reduce persistent inflammation during pneumonia, and reduced amounts of butyrate-producing anaerobic bacteria are associated with an increased risk of developing respiratory tract infections in patients undergoing allogeneic hematopoietic stem cell transplantation [11,12].

In contrast to the increasing interest in the role of the bacterial microbiome during respiratory infections, little is known about the role of other organisms inhabiting the intestine, such as eukaryotic viruses and bacteriophages, jointly called the virome. The relationship between the virome and the host is dynamic and complex, as these micro-organisms have been shown to directly interact with the bacterial microbiome [13,14]. Bacteriophages contribute to the virulence potential of pathogenic bacteria by suppressing antibacterial immunity and possibly preventing clearance of bacterial infection [15]. These interactions between bacteria and bacteriophages might be relevant to commensal intestinal bacteria. Recent studies suggested the importance of intestinal viruses in both intestinal as systemic diseases. For example, decreased viral diversity was associated with the severity of disease of ulcerative colitis, and changes in the virome over time preceded autoimmunity in type I diabetes-susceptible children [16,17]. Despite of the findings linking both the virome and bacteriome to health and disease, the role of these communities during CAP remains unclear.

To characterise the rectal bacterial and viral communities of CAP patients at hospital admission and following discharge, and to determine if microbiota composition at admission correlates with clinical outcomes, we performed an exploratory prospective, observational cohort study in hospitalised CAP patients and controls. The clinical outcomes of interest were time to clinical stability (defined according to the Halm's criteria) [18], a common outcome measure for pneumonia [19], and length of hospital stay. We hypothesized that the composition of the bacteriome and virome would be disrupted during CAP and that co-occurrence signatures of these disturbances, identified using unsupervised factor analysis [20], could be associated with clinical outcomes.

## Methods

### Study design and participants

Participants were recruited as part of the ELDER-BIOME study. Details of recruitment and data collection have been previously published [21]. In short, consecutive patients older than 18 years admitted to a university (Amsterdam UMC, location Academic Medical Centre) or community hospital (BovenIJ hospital) in the Netherlands between October 2016 and July 2018, with the clinical suspicion of a community-acquired pneumonia were included, defined as at least one respiratory symptom (new cough or sputum production, chest pain, dyspnea, tachypnea, abnormal lung examination, or respiratory failure) and one systemic symptom (documented fever or hypothermia, leukocytosis or leukopenia) and had an evident new or progressive infiltrate, consolidation, cavitation, or pleural effusion on chest X-ray or computed tomography scan. Patients exposed to antibiotics within 48 h prior to hospital admission, with the clinical suspicion of an aspiration pneumonia or hospital-associated pneumonia were excluded from study participation. Subjects of comparable age without acute infection, who presented at the outpatient clinic of the Amsterdam UMC, location Academic Medical Centre, served as controls. The ELDER-BIOME study was compiled in accordance with the Declaration of Helsinki and approved by the local institutional review boards (reference: NL57847.018.16). All eligible participants, or their legal representatives, provided written informed consent.

### Procedures

Rectal swabs in Universal Transport Medium (Copan, Murrieta, CA, USA) were collected within 24 h of hospital admission and one month thereafter. This timepoint was selected based on previous studies which have shown that the faecal microbiota remains essentially similar within the first 24 h of antibiotic treatment [22,23]. Rectal swabs were used to increase the likelihood of obtaining a sample within this time window. Rectal swabs at one month following hospital admission were collected at the hospital ward (for inpatients) or during a follow-up visit at the outpatient clinic (for discharged patients). Upon completion of sample collection, bacterial microbiota were characterised by a 16S rRNA gene sequencing targeting the V3-V4 region and viral microbiota were sequenced by virus discovery next-generation sequencing (VIDISCA), using a validated virome-enriched library preparation, both earlier described by our group [24], [25], [26], [27]. Additional details on the 16S rRNA gene sequencing and VIDISCA are provided in the Supplementary Methods.

### Statistical analysis

All analyses were performed using R (version 3.6.0, Vienna, Austria). To investigate patterns of covariation between rectal bacteria and viruses during and following CAP hospitalisation, we employed multi-omics factor analysis (MOFA) [20,25,28]. MOFA captures the principal sources of variation in a small number of inferred factors or signatures. The interpretation of these factors is comparable to the interpretation of the principal components in principal component analysis. In addition, MOFA calculates a weight for all bacterial and viral features in each factor. Features with large weights are highly correlated (positively or negatively) with the factor values. Details on the methods for the assessment of α- and β-diversity, Spearman correlations and the MOFA model are provided in the Supplementary Methods.

We fitted both a linear plateau model (rcompanion package) and natural cubic B-splines (splines package) with 2 internal knots (tertile split) to model the relation between the Factor 1 value and the (log-transformed) values of our clinical outcome of interest. Next, time-to-event analysis was performed by defining two groups of CAP patients at admission based on the optimal cut-point of the MOFA factor value and Shannon index for the time to clinical stability, determined using maximally selected rank statistics [29]. Univariable and multivariable Cox proportional hazard models were used to assess potential predictors of clinical outcomes. The time to clinical stability was one of our clinical outcomes of interest and is defined according to the Halm's criteria, which consist of six clinical parameters (systolic blood pressure ≥90 mmHg, heart rate ≤100 beats/min, respiratory rate ≤24 breaths/min, oxygen saturation ≥90% or arterial oxygen tension ≥60 mmHg, and temperature ≤37•8 °C) [18]. As the dichotomisation of Factor 1 by an optimal cut-point could result in an overfitted model, as multiple candidate cut-points are examined, we calculated adjusted *p*-values using the maxstat package (function maxstat.test) on Factor 1 values below the plateau. Additional detail on the time-to-event analysis is available in the Supplementary Methods.

Data were not normally distributed and are therefore presented as median with interquartile range (IQR). Continuous data were analysed using a Mann-Whitney U or Kruskal-Wallis test, which are robust to unequal sample sizes. False-discovery rate was adjusted for with the Benjamini-Hochberg procedure. The two-tailed level of significance between groups was set at (adjusted) *p*<0•05.

### Role of the funding source

This work was supported by the Netherlands Organization for Scientific Research and the Netherlands Organization for Health Research and Development. The funders had no role in study design, data collection and analysis, data interpretation, decision to publish, or preparation of the manuscript. All authors had access to the data used for analyses and took the decision to submit for publication.

## Results

### Study cohort

64 patients with CAP were enrolled in this study at admission and followed up after one month, in addition to 38 control subjects. Patients who were lost to follow up were excluded. The Consolidated Standards of Reporting Trials flowchart of the study, including details on participant inclusion, follow up and exclusion of specimens is depicted in Supplementary Fig. 1. Patient demographics and clinical characteristics are reported in Table 1. Demographic characteristics, body mass index, prior antibiotic exposure and comorbidities (diabetes, cardiovascular disease, malignancy, gastrointestinal disease and/or chronic renal disease) were comparable between CAP patients and controls. However, CAP patients had a higher prevalence of chronic obstructive pulmonary disease (COPD; *p* = 0•029).

**Table 1:** Clinical characteristics of the Study Population.

	Patients (*n* = 64)	Controls (*n* = 38)	p-value
Age, y, median (IQR)	69 (59•8–77•3)	69 (63•3–74•8)	0•56
Male sex, n (%)	38 (59)	22 (58)	1•00
Ethnicity, Caucasian, n (%)	49 (77)	32 (84)	0•91
BMI, kg/m^2^, median (IQR)	26•1 (23•2–28•4)^a^	26•3 (24•7–28•6)	0•29
Past smoker, n (%)	31 (48)	18 (47)	0•80
Influenza vaccination^b^, n (%)	41 (64)	19 (50)	0•15
Prior antibiotic use^c^, n (%)	10 (16)	2 (5)	0•21
Comorbidities			
COPD, n (%)	18 (28)	3 (8)	0•029
Immunosuppressed^d^, n (%)	18 (28)	4 (10)	0•066
Cardiovascular disease, n (%)	49 (76)	27 (71)	0•70
Diabetes, n (%)	16 (25)	4 (10)	0•13
Malignancy, n (%)	22 (34)	6 (15)	0•071
Neurological disease, n (%)	6 (9)	0 (0)	0•13
Gastrointestinal disease, n (%)	8 (12)	1 (2)	0•18
Chronic renal disease, n (%)	9 (14)	2 (5)	0•29
Chronic medication			
NSAIDs, n (%)	4 (6)	4 (11)	0•69
Antihypertensive agents, n (%)	26 (41)	23 (61)	0•082
Proton pump inhibitors, n (%)	33 (52)	13 (34)	0•13
Oral antidiabetics, n (%)	10 (16)	1 (3)	0•086
Laxatives, n (%)	12 (19)	2 (5)	0•11
Microbiology			
*Streptococcus pneumoniae*, n (%)	11 (17)	••	••
*Haemophilus influenzae*, n (%)	7 (10)	••	••
Influenza virus, n (%)	6 (9)	••	••
Other bacterial pathogen, n (%)	7 (10)	••	••
Other viral pathogen, n (%)	7 (10)	••	••
No causative agent found, n (%)	25 (39)	••	••
Severity of disease and outcome			
PSI class, median (IQR)	4 (3–4)	••	••
qSOFA, median (IQR)	1 (0–1)	••	••
ICU admission, n (%)	5 (8)	••	••
Time to clinical stability^e^, days, median (IQR)	3 (2–6)	••	••
Length of hospital stay, days, median (IQR)	4 (3–7)	••	••

Abbreviations: IQR, interquartile range; CAP, community-acquired pneumonia; COPD, chronic obstructive pulmonary disease; NSAIDs, nonsteroidal anti-inflammatory drugs; PSI, Pneumonia severity index; qSOFA, quick Sequential Organ Failure Assessment; ICU, intensive care unit.

a BMI was assessed in 61 patients. ^b^ Received influenza vaccination in the past year. ^c^ Exposure to oral or systemic antibiotics between 90 days and 48 h prior to admission. ^d^ Immunosuppressive disease was defined as clinically suspected or proven immunodeficiency, the use of immunosuppressive therapy or immunomodulating medication in the past 3 months, including chemotherapy, or the use of more than 10 mg prednisone or equivalent each day for the past 3 months. ^e^ Time to clinical stability was defined according to the Halm's criteria.^18^.

*Rectal bacteriome diversity and composition of patients with CAP differs from controls*

**Fig. 1:**
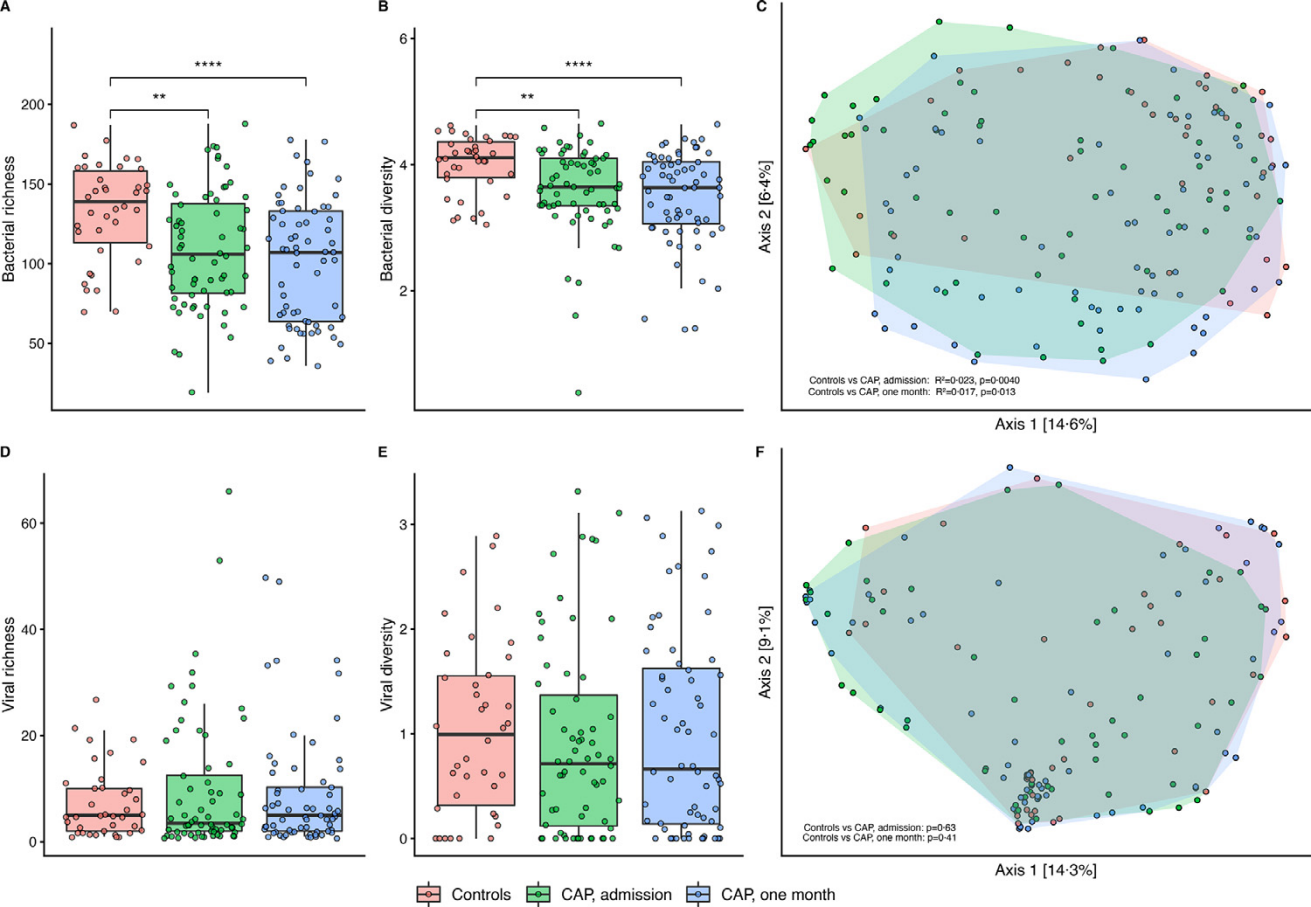
Rectal bacteriome diversity of CAP patients differs from controls. Lower richness *(A)* and α-diversity *(B)* of the bacterial microbiota in CAP patients at admission and after one month, compared to controls. Significant differences are seen in beta-diversity of CAP patients at both timepoints, compared to controls *(C)*. No significant changes in richness, α-diversity or β-diversity of viral microbiota were observed between groups *(D–F).* In the boxplots, the rectangle spans the first quartile to the third quartile (the interquartile range or IQR), the horizontal black line inside the rectangle shows the median, and whiskers above and below the box. Given the non-normal distribution of these data, p-values were calculated using the Wilcoxon rank sum test. β-diversity as depicted by Bray-Curtis dissimilarity index in a principal coordinate analysis (PCoA). Each dot represents a sample, coloured by group. Shaded area spans all samples per group. Dissimilarities in gut microbiota composition (*R*^2^) and *p*-values are determined using permutational multivariate analysis of variance. **= *p*<0•01–0•001; ****= *p*<0•0001.

Analysis of the rectal bacterial microbiome showed a significantly lower bacterial richness (*p* = 0•00,092; Fig. 1A) and α-diversity (*p* = 0•0015; Fig. 1B) in CAP patients at admission, as well as after one month (*p*<0•0001 and *p* = 0•00,012; Fig. 1A,B), compared to controls. In addition, β-diversity of bacterial communities in CAP patients (at both timepoints) differed significantly from controls (*r^2^*=0•023, *p* = 0•0040 for admission, and *r^2^*=0•017, *p* = 0•013 after one month; Fig. 1C).

We found comparable differences in bacterial α- and β-diversity between CAP patients and controls when we excluded participants with antibiotic perturbation in the 90 days up to 48 h prior to hospital admission, and a subgroup analysis of patients and controls without COPD showed similar results as well (see Supplementary Fig. 2). However, this does not rule out the possibility of residual confounding by these or other exposures (e.g. immunosuppressed state).

**Fig. 2:**
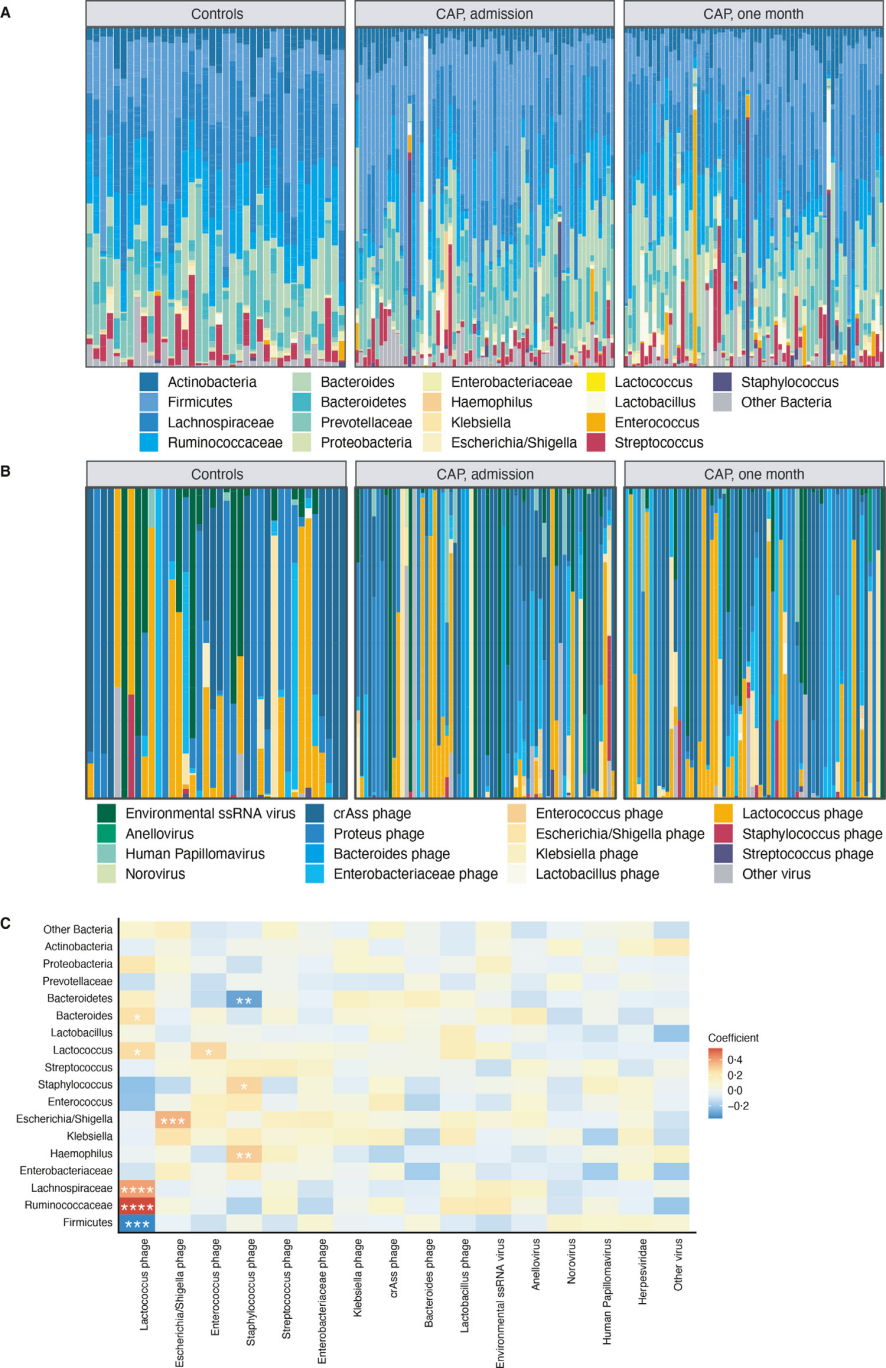
Rectal bacteriome and virome composition of CAP patients differs from controls and co-occur. Relative proportion of sequence reads per sample of bacterial microbiota *(A)* and viral microbiota *(B)*. Heat map of Spearman's rank correlation coefficients between the relative proportions of sequence reads of bacteria and viruses *(C)*. Colours in the heat map represent the *ρ*-values of Spearman correlations (i.e. the strength of the correlation); ‘*’ marks Benjamini-Hochberg corrected p-values <0•05, **= *p*<0•01–0•001; ***= *p*<0•001–0•0001; ****= *p*<0•0001.

No significant changes in richness (*p* = 0•76 for CAP patients at admission compared to controls), α- (*p* = 0•34) nor β-diversity (*p* = 0•63) of viral microbiota were observed (Fig. 1D–F). In depth analysis of bacterial composition revealed personalised patterns, yet Firmicutes and Bacteroides were the predominant phyla in all samples (Fig. 2A). Interestingly, in some CAP patients, especially patients one month after hospital admission, we observed high relative abundances of potentially pathogenic bacteria, such as *Enterococccus* and *Staphylococcus* species (Supplementary Fig. 3).

Viral communities were largely dominated by environmental single stranded (ss) RNA viruses, which mostly represent plant viruses of dietary origin, and bacteriophages of the order Caudovirales, such as cross-assembly (crAss) phage (observed in 45•2% of samples), Enterobacteriaceae phage and Lactococcus phage (both observed in 43•4% of samples) (Fig. 2B and Supplementary Table 1), which are all hosted by commensal intestinal bacteria [30].

To identify potential co-occurrences of rectal bacteria and viruses, we used Spearman correlations of the relative proportion of reads. As expected, the relative abundances of several bacteria - such as *Lactococcus, Escherichia/Shigella* and *Staphylococcus* species - were positively correlated with that of its corresponding phage (Benjamini-Hochberg adjusted *p*-values <0•05; Fig. 2C). Furthermore, the relative abundance of Lactococcus phage was strongly correlated with higher occurrences of Ruminococcaceae (*ρ*=0•55, *p*<0•0001) and Lachnospiraceae (*ρ*=0•40, *p*<0•0001), both families of obligate anaerobic gut commensals, but lower occurrences of other Firmicutes (*ρ*=−0•37, *p* = 0•0001; Fig. 2C). We thus concluded that rectal bacterial diversity of CAP patients differs from controls, yet no significant changes in diversity of viral microbiota are observed.

### Variation in rectal bacteriome and virome composition between CAP patients and controls is captured by unsupervised analysis in Factor 1

To further analyse patterns of covariation between bacterial and viral communities, we used multi-omics factor analysis (MOFA), which enables the unsupervised integration of these communities. Unsupervised multi-omics analyses are powerful tools to unmask potential phenotypes or discover biomarkers that span across biological domains. Moreover, MOFA enables concurrent analysis of the bacteriome and virome composition, as opposed to other methods, such as differential relative abundance or α-diversity. To integrate bacterial and viral communities, MOFA identifies the variability in microbiota composition and uses latent factors or signatures (i.e. sources of variation) to capture this variation. MOFA calculates a value for each sample (the factor values), based on the abundance of specific bacterial and viral taxa within that sample. Hence, MOFA facilitates the unsupervised identification of a limited number of bacterial and viral taxa that drive the variation of the gut microbiome within the cohort.

**Fig. 3:**
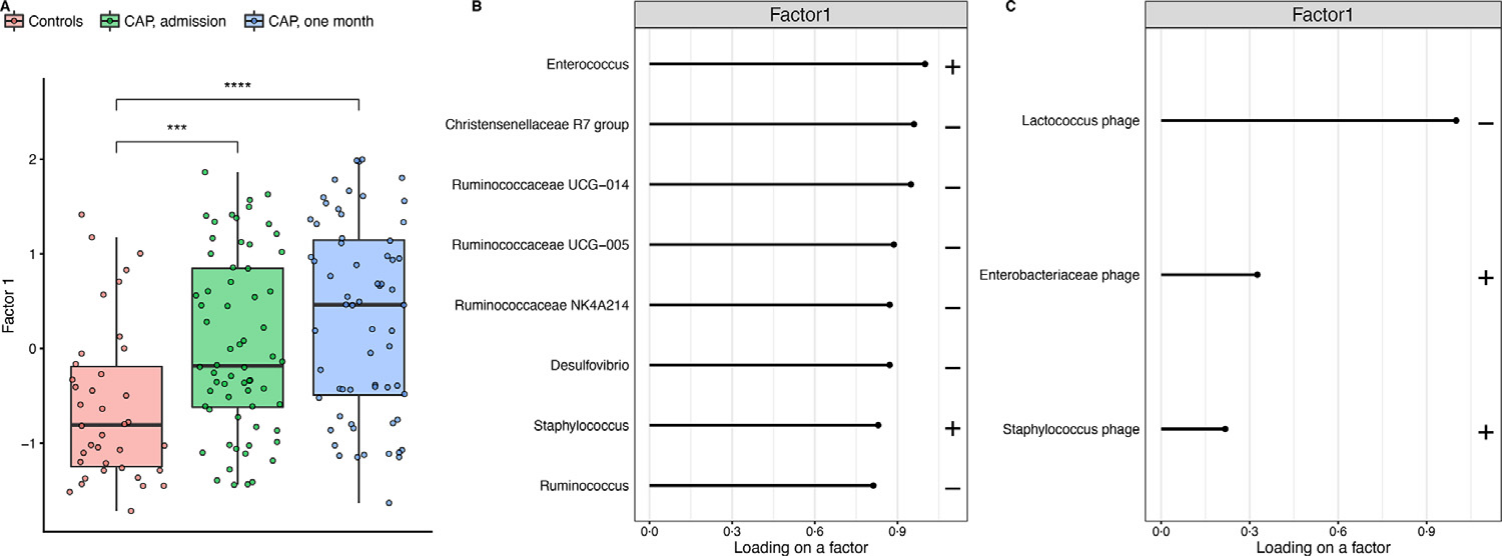
Variation in rectal bacteriome and virome composition between CAP patients and controls is captured by unsupervised analysis in Factor 1. Controls have lower Factor 1 values, a score based on the major source of variation in microbiota composition in our study as identified by MOFA, than CAP patients, both at admission and after one month *(A)*. The bacterial *(B)* and viral *(C)* weights for Factor 1 illustrating which bacteria and viruses are important for the value of Factor 1. The plus sign indicates a positive correlation with the value of Factor 1, whereas the minus sign indicates a negative correlation with the value of Factor 1. The larger the value of the weight (loading), the stronger the association. The weights with the highest values are showed. In the boxplots, the rectangle spans the first quartile to the third quartile (the interquartile range or IQR), the horizontal black line inside the rectangle shows the median, and whiskers above and below the box. Given the non-normal distribution of the data, p-values were calculated using the Wilcoxon rank sum test. ***= *p*<0•001–0•0001; ****= *p*<0•0001.

CAP patients (at both timepoints) had higher values of Factor 1, the major source of variation, than controls (*p*<0•001; Fig. 3A), indicating that Factor 1 captured variation in bacteriome and virome composition associated with CAP. Specifically, Factor 1 represents a signature based on a small number of bacteria and viruses, which is capable of discriminating CAP patients from controls.

**Fig. 4:**
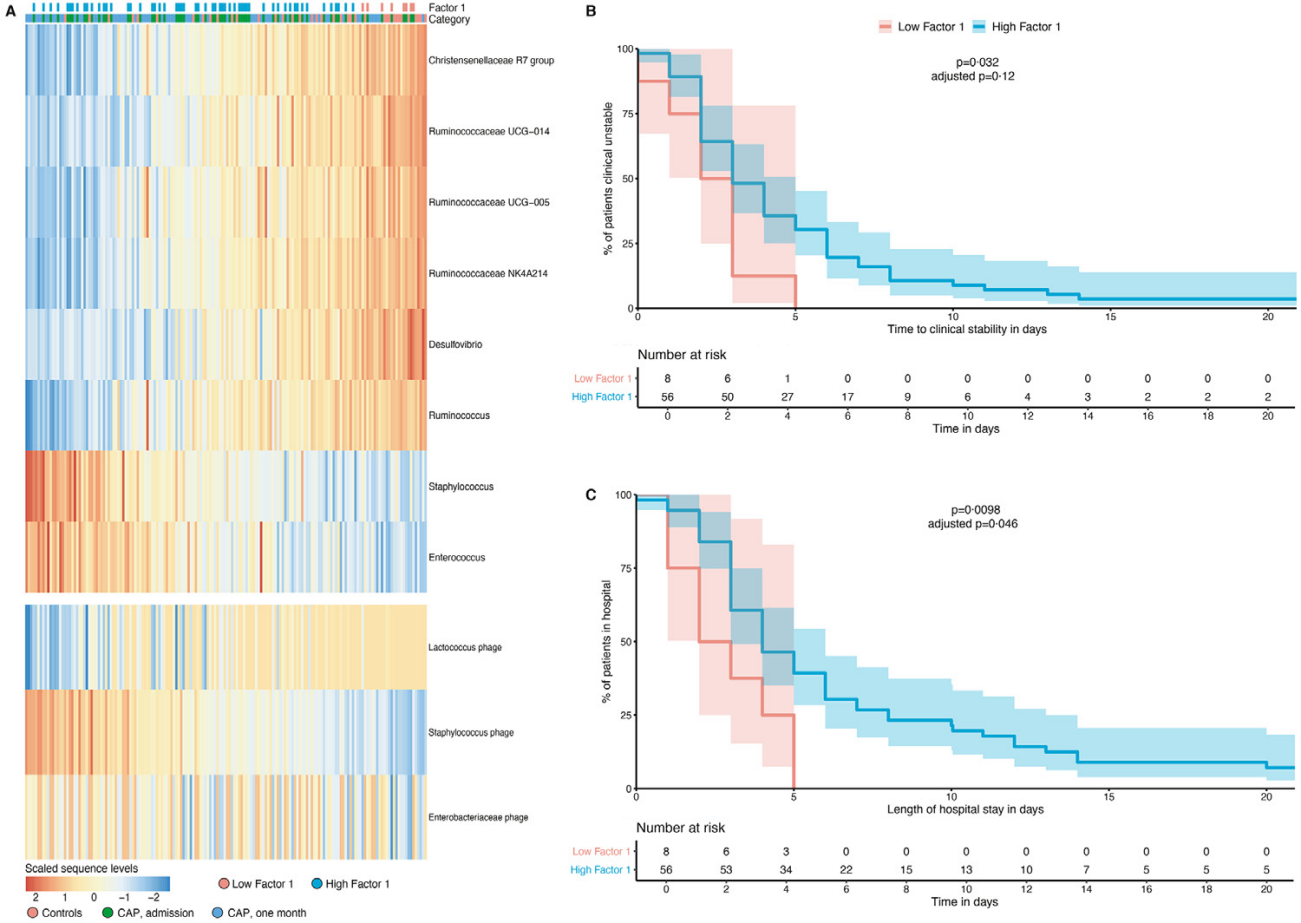
Bacterial and viral microbiota signatures are coupled with clinical outcomes in patients with CAP. Heat map (*A*) of the scaled sequences levels (a normally-distributed denoised representation of the input data generated by MOFA by taking the product of the factors and the weights) for the bacteria and viruses (rows) displayed in Fig. 3B,C, samples are shown in the columns and annotated by category and the value of Factor 1 in CAP patients at admission. Kaplan-Meier plots measuring time to clinical stability (*B*; x-axis; defined according to Halm's criteria) and length of hospital stay (*C*; x-axis) for the value of Factor 1 in CAP patients at admission. The cut-point on the Factor 1 value was chosen using maximally selected rank statistics. CAP patients with a low Factor 1 have lower abundances of, among others, *Enterococcus* and *Staphylococcus* spp and higher abundances of Lactococcus phage and several commensal bacteria, compared to patients with a high Factor 1. Log-rank p-values are significant if *p*<0•05. Adjusted *p*-values (for examining multiple cut-points) are calculated using the maxstat package.

Analysis of the weights - which can be interpreted as a measure of importance of certain bacteria and viruses for the value of a factor - revealed that bacteria positively correlated with Factor 1 were *Enterococcus* and *Staphylococcus* species. Genera within the family Ruminococcaceae (*Ruminococcus*, Ruminococcaceae UCG-014, UCG-005, NK4A214) and other obligate anaerobic commensal gut bacteria (Christensenellaceae R7 group, *Desulfovibrio*) were negatively correlated (Fig. 3B). Viral features that were positively correlated with Factor 1 were Enterobacteriaceae and Staphylococcus phages, while Lactococcus phage was negatively correlated (Fig. 3C). Therefore, these findings indicate that in this cohort, the bacteriome and virome of CAP patients contains higher abundances of *Enterococcus* species among others, and lower abundances of obligate anaerobic commensal gut bacteria as well as Lactococcus phages compared to controls (Fig. 4A).

### Bacterial and viral microbiota signatures are coupled with clinical outcomes in patients with CAP

Because Factor 1 represents changes in rectal bacteriome and virome composition between CAP patients and controls (Fig. 3A), we hypothesized that the microbiota signature represented by this Factor could serve as a marker for a healthy rectal microbiome and correlate with clinical outcomes. Using a linear plateau model and cubic spline analysis, we found that low Factor 1 value is associated with a shorter length of hospital stay until a threshold of Factor 1 is reached (Supplementary Fig. 4).

Next, CAP patients at admission were divided into two groups based on the optimal cut-point value of Factor 1 for the time to clinical stability (Factor 1 = −1.01), chosen using maximally selected rank statistics [29]. Baseline characteristics and severity scores, except ethnicity (*p* = 0•037) and smoking history (*p* = 0•012), did not differ significantly between these two groups (Supplementary Table 2). We first asked if Factor 1 correlated with the time to clinical stability (defined according to Halm's criteria) [18]. Using univariable analyses, we found that a low Factor 1 was associated with a shorter time to clinical stability when compared to patients with a high Factor 1 (HR 0•43; 95% confidence interval (CI) 0•20–0•93, *p* = 0•032; Table 2). These findings indicate that patients with higher abundances of *Enterococcus* species and lower abundances of obligate anaerobic commensal gut bacteria and Lactococcus phages, were less likely to become clinically stable (*p* = 0•032; Fig. 4B). Demographic characteristics and comorbidities can be potential confounders in clinical studies. When we controlled for several potential confounders (age, sex, ethnicity, prior use of antibiotics, COPD, immunocompromised status and severity of disease by pneumonia severity index (PSI)) [31] in a multivariable analysis, the relationship between a high Factor 1 value and time to clinical stability remained significant (HR 0•24, 95% CI 0•09–0•66, *p* = 0•0055; Table 2). We next asked if the value of Factor 1 was associated with the length of hospital stay (LOS), our other outcome of interest. Similarly to the time to clinical stability, a low Factor 1 correlated with shorter LOS when compared to CAP patients with a high Factor (*p* = 0•0098; Fig. 4C). The HR for a favourable outcome (discharge) was 0•37 (95% CI 0•17–0•81, *p* = 0•012; Supplementary Table 3) for patients with a high Factor 1. Moreover, the relationship between the Factor 1 value and LOS remained significant in a multivariable analysis (*p* = 0•00,043; Supplementary Table 3).

**Table 2:** Associations with time to clinical stability in CAP patients at admission.

	Univariable		Multivariable	
Predictor	Hazard Ratio (95% CI)	p-value	Hazard Ratio (95% CI)	*p*-value
Age, y	1•00 (0•99–1•02)	0•616	1•00 (0•98–1•02)	0•955
Male sex	0•92 (0•56–1•52)	0•740	0•83 (0•48–1•44)	0•503
Caucasian ethnicity	0•70 (0•39–1•27)	0•24	0•67 (0.35–1.28)	0•225
Prior antibiotic use^a^	0•79 (0•39–1•60)	0•509	1•01 (0•47–2•18)	0•975
COPD	1•22 (0•70–2•12)	0•488	1•93 (0•92–4•03)	0•082
Immunosuppressed^b^	1•28 (0•74–2•21)	0•385	2•05 (1•03–4•05)	0•040
Severity of disease (PSI)	0•90 (0•71–1•13)	0•351	0•81 (0•63–1•06)	0•12
Microbiota features				
Factor 1, high group	0•43 (0•20–0•93)	0•032	0•24 (0•09–0•66)	0•0055
Shannon diversity index, high group^c^	0•67 (0•40–1•11)	0•118	••	••

Univariable and multivariable Cox proportional hazards models were used to assess potential predictors of time to clinical stability.

Abbreviations: CI, confidence interval; COPD, chronic obstructive pulmonary disease; PSI, Pneumonia severity index.

a Exposure to oral or systemic antibiotics between 90 days and 48 h prior to admission. ^b^ Immunosuppressive disease was defined as clinically suspected or proven immunodeficiency, the use of immunosuppressive therapy or immunomodulating medication in the past 3 months, including chemotherapy, or the use of more than 10 mg prednisone or equivalent each day for the past 3 months. ^c^ Group with a Shannon diversity index > 3•70.

As the dichotomisation of Factor 1 by an optimal cut-point could have resulted in an overfitted model, we calculated adjusted *p*-values for examining multiple candidate cut points using the maxstat package. When we derived an optimal cut point on Factor 1 below the plateau, we found a statistically significant relation between the Factor 1 value and the length of hospital stay (*p* = 0•046; Fig. 4C). Importantly, the association between the Factor1 value and the time to clinical stability was not significant (*p* = 0•12; Fig. 4B) following p-value adjustment for examining multiple candidate cut-points, and thus could be consistent with chance.

Of note, when we used MOFA to capture the variability in only bacterial microbiota - without viral microbiota - there was no significant relationship with time to clinical stability (*p* = 0•094; Supplementary Fig. 5). In addition, α-diversity – an earlier proposed marker for a healthy gut microbiome [32,33] – was not associated with time to clinical stability (*p* = 0•12; Table 2), nor LOS (*p* = 0•16; Supplementary Table 3). Taken together, the findings of this exploratory study suggest that variation in a small number of rectal bacteria and viruses during CAP might reflect a previously unappreciated marker of clinical outcomes.

## Discussion

This prospective observational cohort study shows that alterations of rectal microbiota during CAP not only involve bacteria but extend to viral communities. Moreover, by using a validated unsupervised analysis, we found that disrupted gut microbiota profiles of CAP patients at hospital admission are coupled with altered clinical outcomes. To our knowledge, this exploratory study is the first to characterise both the gut bacteriome and virome during CAP in humans, and the first to demonstrate that alterations of these communities might be associated with clinical outcomes in CAP patients.

We and others have demonstrated in mice that both bacterial and viral respiratory infections are associated with a change in the composition of the bacterial gut microbiota, caused by a combination of physiological and environmental influences [34,35]. For example, respiratory infection-induced anorexia is at least partly responsible for microbiota alterations as food-restricted mice displayed reduced amounts of Lachnospiraceae, a family of obligate anaerobic bacteria capable of producing health-promoting short-chain fatty acids [36]. The present study shows that these associations extend to humans, as patients presenting to the hospital with CAP had bacterial gut communities that markedly differed from those in controls. Their bacteriome was characterised by a loss of commensal bacteria, such as genera belonging to the families Lachnospiraceae and Ruminococcaceae, and higher abundances of *Enterococcus* and *Staphylococcus* species. The loss of commensal bacteria during illness enables the overgrowth of these facultative aerobic pathobionts [7]. The bacteriome remained disturbed one month following hospital admission, indicating that, in line with previous studies [6,24], the impact of hospitalisation and antibiotic exposure on the microbiome is profound and recovery is slow.

In accordance with earlier cohorts, the rectal virome in our study was dominated by dietary plant viruses, crAss phages and other bacteriophages belonging to the order of Caudovirales [37,38]. CrAss phages have recently been linked to Bacteroides species and most other Caudovirales are hosted by commensal intestinal bacteria, such as Firmicutes and Bacteroides [37]. Using Spearman correlations, we observed positive associations between the abundance of several bacteria and their corresponding phage. Despite this dependency of viral communities on their bacterial counterparts and a decrease in bacterial α-diversity during and following CAP, the diversity of the virome in our study remained unchanged. The changes in the bacteriome during CAP may not be sufficient to alter viral communities as earlier studies reported that the correlation between viral α-diversity and bacterial diversity is only mild, and suggested that viral community diversity may not be affected by the use of antibiotics [38,39]. However, the lack of difference in virome composition in this exploratory study could also be explained by the large heterogeneity and individual differences in viral α-diversity and community compositions, or by the limited sensitivity of VIDISCA next-generation sequencing [27]. Future studies with higher sequencing depth, the possibility to differentiate between integrated and non-integrated phages and a larger sample size are needed to further clarify the role of the intestinal virome in pneumonia.

We were able to define two groups of patients with CAP based on the reduction of obligate anaerobic bacteria, overgrowth of potentially pathogenic bacteria and reduced abundance of Lactococcal phages, which had a statistically significant difference in the time to clinical stability and duration of hospitalisation. However, only the latter remained significant following p-value adjustment for examining multiple candidate cut-points.

Although faecal domination by potentially pathogenic bacteria (such as *Enterococcus* species) and a decrease in obligate anaerobes in critically ill patients has been associated with an increased risk for death and complications [40,41], and preclinical studies showed a protective effect of bacterial microbiota against respiratory pathogens [8], [9], [10], we are the first to describe that a rectal microbiota signature may predict clinical outcomes during CAP. This signature, comprising a small number of bacteria and viruses, may serve as a proof-of-concept for the possibilities of microbiota-based prediction of clinical outcomes and, when adequately validated in other cohorts, could potentially inform the future development of a standard multiplex PCR panel used to predict disease severity in CAP.

While transkingdom variation was independently associated with clinical outcomes, there was no significant association between the time to clinical stability and bacterial composition or bacterial α-diversity in our study. Therefore, our findings suggest that the combination of viral and bacterial microbiota markers are potentially better predictors for the severity of disease in respiratory tract infections than changes in bacterial composition alone.

Changes in composition of intestinal microbiota during CAP might not solely be a consequence of disease, but certain obligate anaerobic intestinal bacteria possibly influence the severity of infections. These bacteria produce butyrate, a short-chain fatty acid, that influences alveolar macrophages, enhances activity against respiratory pathogens and potentially reduces persistent lung inflammation during pneumonia [9,11,12,42]. Moreover, bacteriophage induced lysis of bacteria is expected to result in the release of molecules (such as lipopolysaccharide and peptidoglycan) that serve as microbe-associated molecular patterns which prime the innate immune system and increase activity against bacterial pathogens [43], [44], [45]. Therefore, the reduction of anaerobic bacteria capable of producing butyrate combined with altered abundances of several bacteriophages associated with Factor 1 in our study might have resulted in worse outcomes. Although the dichotomisation of Factor 1 could have resulted in an overfitted model, our linear plateau model suggests that a threshold for a healthy microbiota exists and the loss of a critical amount of beneficial intestinal micro-organisms might be associated with worse clinical outcomes. Of note, the association between the Factor1 value and LOS remained significant following p-value adjustment for examining multiple candidate cut-points, whereas the association between the Factor 1 value and the time to clinical stability was not significant following these adjustments.

Future research should elucidate the exact mechanism by which alterations in intestinal microbiota composition, both bacterial and viral, affect the immune system and influence clinical outcomes. Such studies could potentially lead to interventions such as restoration of an altered microbiota or the use of microbiota-derived metabolites to improve outcomes for CAP patients.

This study was designed as a small proof-of-concept study of exploratory nature and has several limitations. First, despite our efforts to address for confounders when comparing CAP patients with controls (an age-matched cohort with comparable demographic characteristics, antibiotic exposure and comorbidities), the described differences might reflect undocumented differences between these groups (e.g. diet and genetic makeup of the host). Moreover, the observational nature of this study and limited sample size limits the possibilities to control for all possible confounders such as comorbidities and medication use.

Second, although we used a validated approach for viral sequencing, the sensitivity of VIDISCA next-generation sequencing reaches 1 E6 viral genome copies/ml and therefore we might not have identified low abundant viruses, and underestimated inter-individual differences in virome α-diversity and composition which could have been identified by other techniques such as total transcriptome sequencing [27,46]. In addition, the analytical approach of VIDISCA did not allow for the discrimination between integrated and non-integrated bacteriophages. Third, we observed that a rectal microbiota signature (Factor 1) was independently associated with clinical outcomes independent of clinical confounders. However, due to the dichotomisation of Factor 1 and the relatively limited sample size these analyses might have been overfitted, which is underscored by the absence of a significant relation between the Factor 1 vale and the time to clinical stability following p-value adjustment for examining multiple candidate cut-points. Fourth, the Factor 1 value is based on a cohort-specific matrix factorization analysis and therefore not generalizable to other patient populations. Consequently, the microbial signatures we identified were not replicated in an independent cohort and warrant confirmation in larger and more diverse patient cohorts, before they can be used to improve triage and patient care in hospital settings.

Finally, although our findings remained significant when controlled for several important confounders, the variation in virome and bacteriome composition at hospital admission for CAP might not influence outcomes, but reflect undocumented differences between CAP patients that lead to worse clinical outcomes.

In conclusion, these results indicate that the combined variation in rectal bacteriome and virome composition could be associated with clinical outcomes in patients with CAP. This exploratory study serves as a proof-of-principle and may inform the future development of a microbiota-based panel used to predict disease severity in CAP.

## Declaration of Competing Interest

All authors report no competing interests.

## Appendix Supplementary materials

Image, application 1
