# Supplementary material for: Rectal bacteriome and virome signatures and clinical outcomes in community-acquired pneumonia: An exploratory study
Source: eClinicalMedicine. 2021 Aug 12;39:101074. doi: 10.1016/j.eclinm.2021.101074 (PMC8478680; doi:10.1016/j.eclinm.2021.101074)
Supplement: Supplementary file 1 [file mmc1.docx]

**Rectal bacteriome and virome signatures are coupled with clinical outcomes in community-acquired pneumonia: an exploratory study**

**Supplementary Material**

Robert F.J. Kullberg, Floor Hugenholtz, Xanthe Brands, Cormac M. Kinsella, Hessel Peters-Sengers, Joe M. Butler, Martin Deijs, Michelle Klein, Daniël R. Faber, Brendon P. Scicluna, Tom van der Poll, Lia van der Hoek, W. Joost Wiersinga^*^, Bastiaan W. Haak^*^

^*^These authors contributed equally to this work

**Supplementary Methods**

**Additional details on 16S rRNA sequencing**

DNA was extracted from the rectal swabs in one run using a repeated bead beating protocol. After DNA purification using the Maxwell RSC Whole Blood DNA Kit (Promega, Madison, WI, USA), 16S rRNA gene amplicons were generated using a single step PCR protocol targeting the V3-V4 region. The libraries were sequenced using a Miseq platform (Illumina, San Diego, CA, USA) using V3 chemistry with 2x251 cycles. Amplified Sequence Variants (ASVs) were inferred for each sample individually with a minimum abundance of 4 reads.^1^ Two positive controls, consisting of genomic material of 55 different strains, were included to evaluate performance. Contaminants were identified using the package decontam, manually scrutinized (frequency method) and subsequently, together with known lab specific contaminants, removed from the dataset. In total, seven contaminating OTUs were removed. Bacterial taxonomy was assigned using the RDP classifier and SILVA 16S ribosomal database.^2,3^

**Additional details on VIDISCA**

Viral microbiota were sequenced by VIDISCA using a validated virome-enriched library preparation.^4–7^ Rectal swab Universal Transport Medium was centrifuged to remove cells and debris. The supernatant was treated with TURBO™ DNase (Thermo Fisher Scientific, Waltham, MA, USA) to degrade residual bacterial or chromosomal DNA. Nucleic acids were extracted using the Boom method which is described in detail elsewhere.^8^ In short, following degradation of bacterial and chromosomal DNA silica particles were added (as solid nucleic acids carrier) to bind the nucleic acids, forming complexes which could be rapidly sedimented by centrifugation These complexes are washed twice with a guanidium thiocyanate containing washing buffer, twice with 70% ethanol, and once with acetone. The complexes are dried and subsequently the nucleic acids are eluted in an aqueous low-salt buffer.

Following the nucleic acids extraction, reverse transcription with non-ribosomal random hexamers was performed.^9^ Second strand synthesis was performed with 5 U Klenow polymerase (3' - 5' exo-; New England Biolabs, Ipswich, MA, USA) and 5 U of RNAseH (New England Biolabs) followed by a phenol chloroform extraction and ethanol precipitation. DNA was digested with MseI restriction enzyme (10U, T^TAA; New England Biolabs) and ligated to adapters containing a sample identifier sequence. Prior to a 28-cycle PCR using adaptor-annealing primers, small DNA fragments were removed by size selection with Agencourt AMPure XP Beads (Beckman Coulter, Brea, CA, USA). Next, Beads were used to select DNA-strands with a length ranging between 150 and 550 nucleotides. To quantify DNA length and concentration, libraries were analysed using the Bioanalyzer (High Sensitivity Kit, Agilent Genomics, Santa Clara, CA, USA) and Qubit Fluorometer (dsDNA Qubit kit, Thermo Fisher Scientific), respectively. Sample libraries were pooled at the equimolar concentration and 50 pmol DNA of the pool was clonally amplified on beads, using the Ion Chef System (Thermo Fisher Scientific), and sequenced on the Ion PGMTM System (Thermo Fisher Scientific) with the ION 316 Chip (400 bp read length and two million sequences expected per run).

VIDISCA reads were aligned using the SILVA SSU V132 database,^3^ the RefSeq viral genomes database and BWA-MEM to a custom sequence database consisting of human reference genome (hg38).^10^ The PathoID module (PathoScope 2.0) was used to reassign reads with multiple alignments to the best hit.^11,12^ Viral candidates were retained as hit when they could be aligned back to the reference database with a nucleotide identity of at least 90% for 100 base pairs. To ensure that all known eukaryotic viruses were detected with this approach, all reads that remained unmapped in the BWA-MEM step were analysed with a separate virus discovery bioinformatic pipeline, as earlier described.^5,6^

**Additional details on the assessment of α- and β-diversity, and Spearman correlations**

The α-diversity - indicative of the number of unique taxa and their proportion within one sample - for each individual was assessed by calculating the Shannon index and Observed Taxa Richness, with the phyloseq package on a rarefied dataset for the bacterial microbiota and on an unrarefied dataset for viral microbiota.^13^ β-diversity - indicative of how different taxa are distributed within samples and thereby the dissimilarity among samples - was assessed with the Bray-Curtis dissimilarity index and visualized using principal coordinate analysis with the phyloseq and microbiome package.^13,14^ This allows the visualization of the two dimensions (axis) explaining the most variability. Differences in β-diversity among groups were assessed by permutational multivariate analysis of variance, using the vegan package (function ‘adonis’).^15^ The obtained R^2^ gives the proportion of variability in microbiota composition (as observed in the entire dissimilarity matrix) that can be attributed to the studied variable (the three groups).

To identify statistically significant relationships between bacterial and viral microbiota, Spearman correlations were calculated on the relative proportions of reads of bacteria and viruses, using the microbiome package.^14^ Spearman correlations detect more complicated relationships than other metrics, such as Pearson correlations, and otherwise might go undetected.

**Additional details on the MOFA model**

The input to the MOFA model was a list of two matrices with matching samples. One matrix represented bacterial ASVs, the other matrix represented viral sequences. Bacterial ASVs were required to have a minimum of ten ASVs observed in at least one percent of the dataset. The number of bacterial sequences were scaled using a centralized-log-ratio after adding a pseudocount, which has shown to be effective in normalizing compositional data.^16,17^ The number of viral sequences were normalized by total-sum scaling (microbiome package) and subsequently scaled using a centralized-log-ratio after adding a pseudocount.^16^

MOFA captures the principal sources of variation in bacterial and viral microbiota composition via a small number of inferred factors (i.e. sources of variation), described in detail elsewhere.^18,19^ The interpretation of these factors is comparable to the interpretation of the principal components in principal component analysis. After model fitting, the learnt factors enabled a variety of downstream analyses: 1) Weights and loadings were visualized. The model learns a weight for every feature in each factor, which can be interpreted as a measure of feature importance. Features with large weights (in absolute value) are highly correlated (positively or negatively) with the factor values. 2) A compressed low-dimensional representation of the data was generated by taking the product of the factors and the weights. Thereby, MOFA reconstructs a normally-distributed denoised representation of the input data which especially facilitates the visualization of sparse readouts.

**Additional details on the time-to-event analysis**

To perform the time-to-event analysis we divided the CAP patients at admission into two groups, based on the optimal cut-point of the MOFA factor value and Shannon index for the time to clinical stability, determined using maximally selected rank statistics which provides the cut point that corresponds to the most significant relation with the outcome.^20^ Potential predictors of clinical outcomes (age, sex, ethnicity, Pneumonia Severity Index,^21^ chronic obstructive pulmonary disease, and microbiota features) were assessed using univariate and multivariate Cox proportional hazards models, with both time to clinical stability (defined according to the Halm’s criteria^22^) and LOS as outcomes measures. The proportional hazards assumption of all covariates was checked using scaled Schoenfeld residuals (function ‘cox.zph’, survival package). A p-value <0·05 was considered as a violation of the proportionality. For continuous variables (age and pneumonia severity index) the assumption of linearity was checked by plotting the Martingale residuals. Kaplan-Meier estimates were calculated and differences in clinical outcomes were assessed using the log-rank test.

**Supplementary References**

1. Callahan BJ, McMurdie PJ, Rosen MJ, Han AW, Johnson AJA, Holmes SP. DADA2: High-resolution sample inference from Illumina amplicon data. *Nat Methods* 2016; **13**(7): 581–583.

2. Wang Q, Garrity GM, Tiedje JM, Cole JR. Naive Bayesian Classifier for Rapid Assignment of rRNA Sequences into the New Bacterial Taxonomy. *Appl Environ Microbiol* 2007; **73**: 5261–7.

3. Quast C, Pruesse E, Yilmaz P, et al. The SILVA ribosomal RNA gene database project: Improved data processing and web-based tools. *Nucleic Acids Research* 2013; **41**: D590-6.

4. Kinsella CM, Deijs M, van der Hoek L. Enhanced bioinformatic profiling of VIDISCA libraries for virus detection and discovery. *Virus Research* 2019; **263**: 21–26.

5. Edridge AWD, Deijs M, van Zeggeren IE, Kinsella CM, Jebbink MF, Bakker M, van de Beek D, Brouwer MC, van der Hoek L. Viral metagenomics on cerebrospinal fluid. *Genes* 2019; **10**(5): 332.

6. de Vries M, Oude Munnink BB, Deijs M, et al. Performance of VIDISCA-454 in feces-suspensions and serum. *Viruses* 2012; **4**: 1328–1334.

7. van der Hoek L, Pyrc K, Jebbink MF, et al. Identification of a new human coronavirus. *Nature Medicine* **2004**; 10: 368–373.

8. Boom R, Sol JA, Salimans MMM, Jansen CL, Wertheim-Van Dillen PME, van der Noordaa J. Rapid and Simple Method for Purification of Nucleic Acids. *J Clin Microbiol* 1990; **28**(3): 495-503.

9. Endoh D, Mizutani T, Kirisawa R, et al. Species-independent detection of RNA virus by representational difference analysis using non-ribosomal hexanucleotides for reverse transcription. *Nucleic Acids Research* **2005**; 33: 1–11.

10. Li H. Aligning sequence reads, clone sequences and assembly contigs with BWA-MEM. 2013. <http://arxiv.org/abs/1303.3997>. Date last updated: May 26 2013. Date last accessed: January 19 2021.

11. Hong C, Manimaran S, Shen Y, et al. PathoScope 2.0: A complete computational framework for strain identification in environmental or clinical sequencing samples. *Microbiome* 2014; **2**: 33.

12. Byrd AL, Perez-Rogers JF, Manimaran S, et al. Clinical PathoScope: Rapid alignment and filtration for accurate pathogen identification in clinical samples using unassembled sequencing data. *BMC Bioinformatics* 2014; **15**(1): 262.

13. McMurdie PJ, Holmes S. Phyloseq: An R Package for Reproducible Interactive Analysis and Graphics of Microbiome Census Data. *PLoS ONE* 2013; **8**(4): e61217.

14. Lahti L, Shetty S. microbiome R package. 2017. <http://microbiome.github.io>. Date last accessed: January 19 2021.

15. Oksanen J, Blanchet FG, Friendly M. vegan: Community Ecology Package. R package version 2.5-6. 2019. <https://CRAN.R-project.org/package=vegan>. Date last accessed: January 19 2021.

16. Aitchison J. The Statistical Analysis of Compositional Data. *Journal of the Royal Statistical Society Series B (Methodological)* 1982; **44**(2): 139–177

17. Gloor GB, Macklaim JM, Pawlowsky-Glahn V, Egozcue JJ. Microbiome datasets are compositional: And this is not optional. *Frontiers in Microbiology* 2017; **8**: 2224

18. Argelaguet R, Velten B, Arnol D. Multi-Omics Factor Analysis-a framework for unsupervised integration of multi-omics data sets. *Mol Syst Biol* 2018; **14**: e8124

19. Haak BW, Argelaguet R, Kinsella CM, et al. Integrative transkingdom analysis of the gut microbiome in antibiotic perturbation and critical illness. *mSystems* 2021; **6**(2): e01148-20.

20. Hothorn T, Berthold L. On the exact distribution of maximally selected rank statistics. *Computational Statistics & Data Analysis* 2003; **43**: 121–137

21. Fine MJ, Auble TE, Yealy DM, et al. A prediction rule to identify low-risk patients with community-acquired pneumonia. *New Engl J Med* 1997; **336**(4): 243-50.

22. Halm EA, Fine MJ, Marrie TJ, et al. Time to Clinical Stability in Patients Hospitalized With Community-Acquired Pneumonia Implications for Practice Guidelines. *JAMA* 1998; **279**(18): 1452-7.

**Supplementary Table 1. Presence of viruses in samples**

|  | **Controls (n=38)** | **CAP, admission (n=64)** | **CAP, one month (n=64)** | **Total (n=166)** |
| --- | --- | --- | --- | --- |
|  | n (%) | n (%) | n (%) | n (%) |
| **Environmental ssRNA virus** | 16 (42) | 20 (31) | 18 (28) | 54 (32·5) |
| **Anellovirus** | 0 (0) | 1 (2) | 1 (2) | 2 (1·2) |
| **Human Papillomavirus** | 3 (8) | 8 (13) | 6 (9) | 17 (10·2) |
| **Norovirus** | 0 (0) | 1 (2) | 0 (0) | 1 (0·6) |
| **crAss phage** | 14 (37) | 32 (50) | 29 (45) | 75 (45·2) |
| **Proteus phage** | 27 (71) | 40 (63) | 37 (58) | 104 (62·7) |
| **Bacteroides phage** | 0 (0) | 5 (8) | 3 (5) | 8 (4·8) |
| **Enterobacteriaceae phage** | 16 (42) | 25 (39) | 31 (48) | 72 (43·4) |
| **Enterococcus phage** | 0 (0) | 6 (9) | 3 (5) | 9 (5·4) |
| **Escherichia/Shigella phage** | 6 (16) | 15 (23) | 14 (22) | 35 (21·1) |
| **Klebsiella phage** | 1 (3) | 3 (5) | 1 (2) | 5 (3·0) |
| **Lactobacillus phage** | 1 (3) | 1 (2) | 2 (3) | 4 (2·4) |
| **Lactococcus phage** | 21 (55) | 22 (34) | 29 (45) | 72 (43·4) |
| **Staphylococcus phage** | 3 (8) | 6 (9) | 7 (11) | 16 (9·6) |
| **Streptococcus phage** | 5 (13) | 4 (6) | 3 (5) | 12 (7·2) |
| **Other virus** | 4 (11) | 11 (17) | 9 (14) | 24 (14·5) |

Abbreviations: ss: single stranded; crAss: cross-assembly

**Supplementary Table 2. Demographics and clinical characteristics of CAP patients at admission, grouped by Factor 1.**

|  | **Low Factor 1 (n=8)** | **High Factor 1 (n=56)** | **p-value** |
| --- | --- | --- | --- |
| **Age, y, median (IQR)** | 79 (74·5-80·3) | 68 (58·3-76·0) | 0·067 |
| **Male sex, n (%)** | 6 (75) | 32 (57) | 0·56 |
| **Ethnicity, Caucasian, n (%)** | 5 (63) | 44 (79) | 0·037 |
| **BMI, kg/m^2^, median (IQR)** | 26·3 (26·1-27·8) | 25·9 (22·9-28·5)^1^ | 0·26 |
| **Past smoker, n (%)** | 6 (75) | 25 (45) | 0·012 |
| **Influenza vaccination^2^, n (%)** | 6 (75) | 35 (63) | 0·14 |
| **Prior antibiotic use^3^, n (%)** | 0 (0) | 10 (18) | 0·44 |
| **Comorbidities** |  |  |  |
| COPD, n (%) | 1 (13) | 17 (30·4) | 0·53 |
| Immunosuppressed^4^, n (%) | 0 (0) | 18 (32) | 0·14 |
| Cardiovascular disease, n (%) | 7 (88) | 42 (75) | 0·74 |
| Diabetes, n (%) | 1 (13) | 15 (27) | 0·66 |
| Malignancy, n (%) | 2 (25) | 20 (36) | 0·84 |
| Neurological disease, n (%) | 1 (13) | 5 (9) | 1·00 |
| Gastrointestinal disease, n (%) | 1 (13) | 7 (13) | 1·00 |
| Chronic renal disease, n (%) | 2 (25) | 7 (13) | 0·68 |
| **Severity of disease and outcome** |  |  |  |
| PSI class, median (IQR) | 4 (4-4) | 4 (3-4) | 0·33 |
| qSOFA, median (IQR) | 1 (0-1) | 1 (0-1) | 0·72 |
| ICU admission, n (%) | 1 (13) | 4 (7) | 1·00 |
| Time to clinical stability^5^, days, median (IQR) | 3 (2-3) | 3 (2-6) | ·· |
| Length of hospital stay, days, median (IQR) | 3 (2-4) | 4 (3-8) | ·· |

Abbreviations: IQR: interquartile range; CAP: community-acquired pneumonia; COPD: chronic obstructive pulmonary disease; PSI: Pneumonia severity index; qSOFA: quick Sequential Organ Failure Assessment; ICU: intensive care unit.

^1^ BMI was assessed in 53 out of 56 patients with High Factor 1. ^2^ Received influenza vaccination in the past year. ^3^ Exposure to oral or systemic antibiotics between 90 days and 48 hours prior to admission. ^4^ Immunosuppressive disease was defined as clinically suspected or proven immunodeficiency, the use of immunosuppressive therapy or immunomodulating medication in the past 3 months, including chemotherapy, or the use of more than 10 mg prednisone or equivalent each day for the past 3 months. ^5^ Time to clinical stability was defined according to the Halm’s criteria.

**Supplementary Table 3. Associations with length of hospital stay in CAP patients at admission**

|  | **Univariate** | | **Multivariate** | |
| --- | --- | --- | --- | --- |
| **Predictor** | **Hazard Ratio (95% CI)** | **p-value** | **Hazard Ratio (95% CI)** | **p-value** |
| **Age, years** | 1·00 (0·99-1·02) | 0·914 | 1·00 (0·98-1·02) | 1·00 |
| **Male sex** | 1·12 (0·68-1·86) | 0·651 | 1·06 (0·62-1·81) | 0·83 |
| **Caucasian ethnicity** | 0·87 (0·48-1·57) | 0·651 | 1·05 (0·55-1·99) | 0·89 |
| **Prior antibiotic use^1^** | 0·60 (0·29-1·22) | 0·154 | 0·80 (0·37-1·74) | 0·57 |
| **COPD** | 1·15 (0·66-2·00) | 0·625 | 2·21 (1·08-4·53) | 0·030 |
| **Immunosuppressed^2^** | 1·52 (0·87-2·67) | 0·141 | 2·80 (1·38-5·72) | 0·0046 |
| **Severity of disease (PSI)** | 0·90 (0·73-1·12) | 0·335 | 0·71 (0·54-0·95) | 0·021 |
| **Microbiota features** |  |  |  |  |
| Factor 1, high group | 0·37 (0·17-0·81) | 0·012 | 0·15 (0·05-0·43) | 0·00043 |
| Shannon diversity index, high group^3^ | 0·69 (0·41-1·12) | 0·158 | ·· | ·· |

Univariate and multivariate Cox proportional hazards models were used to assess potential predictors of length of hospital stay.

Abbreviations: CI, confidence interval; COPD, chronic obstructive pulmonary disease; PSI, Pneumonia severity index.

^1^ Exposure to oral or systemic antibiotics between 90 days and 48 hours prior to admission.

^2^ Immunosuppressive disease was defined as clinically suspected or proven immunodeficiency, the use of immunosuppressive therapy or immunomodulating medication in the past 3 months, including chemotherapy, or the use of more than 10 mg prednisone or equivalent each day for the past 3 months. ^3^ Group with a Shannon diversity index > 3·70.

**Supplementary Figure 1**

**Supplementary Figure 1. Flowchart of cases and controls inclusion**

Consolidated Standards of Reporting Trials flow diagram of the screening, inclusion and analysis of cases and controls in a 2:1 ratio.

**Supplementary Figure 2**

**
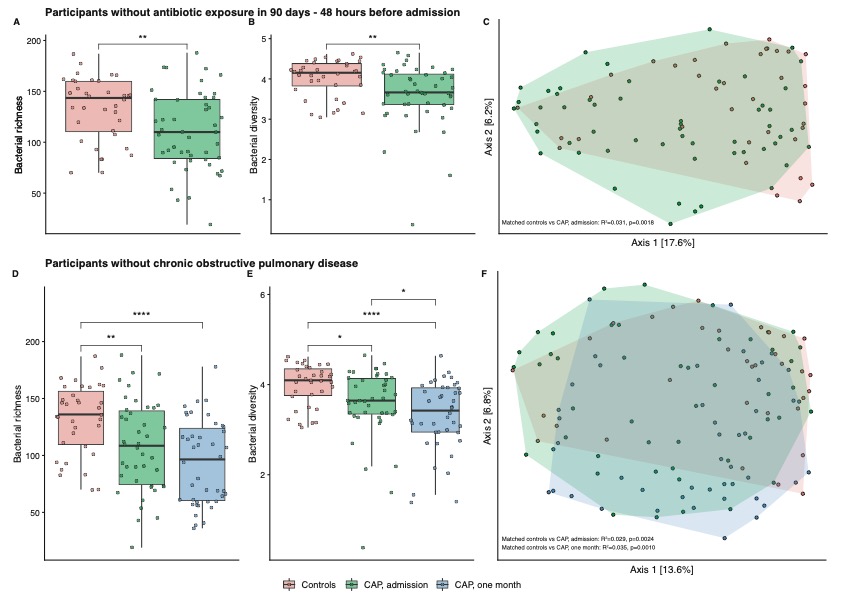
**

**Supplementary Figure 2. Subgroup analyses of rectal bacteriome diversity of CAP patients and controls**

Additional analyses after excluding participants (n=12) with antibiotic exposure in the 48 hours – 90 days before admission confirmed the differences in bacterial richness (A), α-diversity (B) and β-diversity (C) between CAP patients (n=54) and controls (n=36). Significant differences are seen in bacterial richness (D), α-diversity (E) and β-diversity (F) of CAP patients at both timepoints, compared to controls, after excluding patients (n=18) and controls with chronic obstructive pulmonary disease (n=3).

In the boxplot, the rectangle spans the first quartile to the third quartile (the interquartile range or IQR), the horizontal black line inside the rectangle shows the median, and whiskers above and below the box. Given the non-normal distribution of the data, p-values were calculated using the Wilcoxon rank sum test. β-diversity as depicted by Bray-Curtis dissimilarity index in a principal coordinate analysis (PCoA). Each dot represents a sample, coloured by group. Shaded area spans all samples per group. Dissimilarities in gut microbiota composition (R^2^) and p-values are determined using permutational multivariate analysis of variance. *= p<0·05-0·01; **= p<0·01–0·001; ****= p<0·0001.

**Supplementary Figure 3**

**Supplementary Figure 3. High relative abundances of potentially pathogenic bacteria**

A few CAP patients at admission and some patients one month thereafter showed high relative abundances of *Streptococcus*, *Staphylococcus* and *Enterococcus* spp. The bar plot shows the relative proportion of sequence reads per sample.

**Supplementary Figure 4**


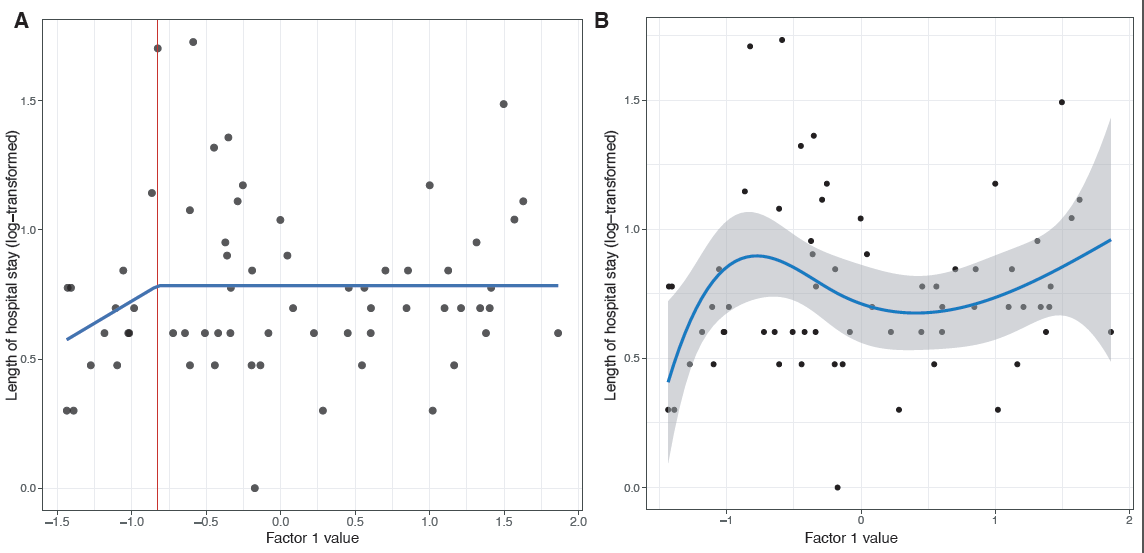


**Supplementary Figure 4. Low Factor 1 values are associated with a shorter length of hospital stay until a threshold of Factor 1 is reached.** Linear plateau model of the log-transformed length of hospital stay by the Factor 1 value. Red line indicates the critical value of Factor 1 (i.e. where the plateau is reached) (A). Log-transformed length of hospital stay by the Factor 1 value with the B-spline regression line with 4 degrees of freedom. Shaded areas indicate the 95% confidence interval (B).

**Supplementary Figure 5**

**Supplementary Figure 5. No correlation between variation in rectal bacteriome and the time to clinical stability**

Kaplan-Meier plots measuring time to clinical stability (x-axis; defined according to Halm’s criteria) for the value of Factor 1 in CAP patients at admission, based on the bacterial microbiota only. Log-rank p-values are significant if p<0·05.
